# Prognoses, outcomes, and clinicopathological characteristics of very elderly patients with hepatocellular carcinoma who underwent hepatectomy

**DOI:** 10.1186/s12957-020-01899-4

**Published:** 2020-06-10

**Authors:** Shingo Shimada, Toshiya Kamiyama, Tatsuya Orimo, Akihisa Nagatsu, Yoh Asahi, Yuzuru Sakamoto, Hirofumi Kamachi, Akinobu Taketomi

**Affiliations:** Department of Gastroenterological Surgery I, Hokkaido University Graduate School of Medicine, North 15-West 7, Kita-Ku, Sapporo, Hokkaido 060-8638 Japan

**Keywords:** Elderly, Hepatectomy, Hepatocellular carcinoma

## Abstract

**Objectives:**

The aim was to evaluate the prognostic factors, clinicopathological characteristics, and surgical outcomes after hepatectomy in very elderly patients with hepatocellular carcinoma (HCC).

**Methods:**

We analyzed 796 patients with HCC from 2000 to 2017. Patients aged 80 years or older were classified into the very elderly group (group VE; *n* = 49); patients younger than 80 years old and aged 65 years or older were classified into the elderly group (group E; *n* = 363), and patients younger than 65 years old were classified into the young group (group Y; *n* = 384). We investigated the prognoses, clinicopathological characteristics, and surgical outcomes after hepatectomy.

**Results:**

The number of surgical procedures and outcomes, including morbidities, was not significantly different. Groups VE, E, and Y showed similar prognoses in terms of both survival and recurrence. In group VE, prothrombin activity (PA) < 80% and PIVKA-II ≥ 400 mAU/ml were unfavorable factors for survival, and PIVKA-II ≥ 400 mAU/ml and the presence of portal venous invasion (PVI), hepatic venous invasion, and fibrosis were unfavorable factors for recurrence. In group E, ChE < 180 IU/l, AFP ≥ 20 ng/ml, tumor size ≥ 10 cm, and the presence of multiple tumors, PVI, and hepatic venous invasion (HVI) were unfavorable factors for survival, and ChE < 180 IU/l, tumor size ≥ 10 cm, and the presence of multiple tumors, PVI, and HVI were unfavorable factors for recurrence. In group Y, AFP ≥ 20 ng/ml, the presence of multiple tumors, poor differentiation, PVI, HVI, and blood loss ≥ 400 ml were unfavorable factors for survival, and PA < 80%, albumin < 3.5 g/dl, AFP ≥ 20 ng/ml, tumor size ≥ 10 cm, and the presence of multiple tumors, poor differentiation, and PVI were unfavorable factors for recurrence.

**Conclusions:**

Tumor factors might have limited influence on the prognosis of very elderly patients, and liver function reserve might be important for the long-term survival of very elderly patients. Hepatectomy can be performed safely, even in very elderly patients. Hepatectomy should not be avoided in very elderly patients with HCC if patients have a good general status because these patients have the same prognoses as nonelderly individuals.

## Background

It is estimated that 18.1 million new cancer cases and 9.6 million cancer deaths occurred in 2018 worldwide [1]. Liver cancer is the seventh most frequent type of cancer with an estimated 841,080 cases per year and the second leading cause of cancer-related death; liver cancer is responsible for approximately 781,631 deaths per year [1]. Hepatocellular carcinoma (HCC) has a poor prognosis and accounts for 70–85% of primary liver cancers [2]. The incident rate of liver cancer in Japan was 23.8% per 100,000 men and 8.6% per 100,000 women [3]. According to the report from the Liver Cancer Study Group of Japan, 6940 liver resections and 122 liver transplantations for HCC occurred between 2008 and 2009 [4]. The surgical mortality rate was 0.4% (30/7062).

The size of the aging population is increasing worldwide. According to a report by the Ministry of Health, Labour and Welfare, the number of people aged 80 years or older was 10.35 million in 2016, which represented 8.3% of the entire Japanese population at the time [5]. The report also states that the average expected life span of 80-year-old individuals is 8.95 years in men and 11.84 years in women [6].

In general, treatment options for HCC include surgical resection; ablation, such as radiofrequency ablation; transcatheter arterial chemoembolization (TACE); hepatic arterial infusion (HAI); liver transplantation; and molecular targeting drugs, such as sorafenib, lenvatinib, regorafenib, and ramucirumab. Furthermore, immunotherapy, i.e., dendritic cell vaccination, may play a critical role as an alternative option in the treatment of advanced HCC patients in whom these traditional therapeutic modalities cannot be applied [7, 8]. However, curative hepatectomy for HCC is a useful method for achieving long-term survival [9]. Thus, the opportunity to treat very elderly people with HCC with hepatectomy has been increasing. Elderly patients have frequent systemic comorbidities due to deteriorating organs and musculoskeletal and cardiovascular functions due to aging [10]. In terms of gastroenterological surgery, hepatectomy is a highly invasive surgical procedure with a high morbidity rate [11]. Surgeons should consider the balance between advantages and disadvantages of the procedure for elderly patients. In addition, various cancer types show different characteristics and prognoses in aging populations. Lung [12], prostate [13], and thyroid [14] cancers show poorer prognoses in elderly patients compared with young patients. In contrast, gastric [15], colorectal [16], and breast [17] cancers show poorer prognoses in young patients compared with elderly patients. There have been some reports concerning HCC; however, the conclusions of these reports are controversial [18, 19].

In this study, we evaluated the prognostic factors and clinicopathological characteristics of patients with HCC aged 80 years or older and compared these patients to those younger than 80 years old after hepatectomy. We also investigated surgical outcomes after hepatectomy.

## Methods

### Patients

Between January 2000 and December 2017, 796 consecutive patients with HCC underwent primary liver resection at the Gastroenterological Surgery I Unit of the Hokkaido University Hospital in Sapporo, Japan. We divided the patients into three groups: patients aged 80 years or older were classified into the very elderly group (group VE; *n* = 49, 6.2%), patients younger than 80 years old and aged 65 years or older into the elderly group (group E; *n* = 363, 45.6%), and patients younger than 65 years old into the young group (group Y; *n* = 384, 48.2%). We compared the prognoses in terms of survival and recurrence, clinicopathological characteristics, and surgical outcomes after hepatectomy between these groups. We defined HBs-Ag positive as HBV and HCV-Ab positive as HCV.

This study was approved by the Hokkaido University Hospital Voluntary Clinical Study Committee (approval 018-0304; 5/Apr/2019) and was performed in accordance with the Helsinki Declaration guidelines. Informed consent was obtained in the opt-out form on the website of Hokkaido University Hospital.

### Hepatectomy

The indications for hepatic resection were as follows: patients with a performance status score between 0 and 2, patients with an American Society of Anesthesiologists (ASA) grade between 1 and 3, patients who were not senile, and patients whose comorbidities were controlled. Patients with or suspected to have ischemic heart disease or cardiac failure were assessed by cardiologists. The type of surgical procedure was usually determined based on the patients’ liver function reserve, i.e., according to the results of the indocyanine green retention test at 15 min (ICGR15) [20]. Anatomical resection was performed for patients with an ICGR15 result less than 25% in principle. However, in some cases, ICGR15 might not represent accurate liver function due to a portosystemic shunt and inconsistent blood collection times [21]. Therefore, if severe cirrhosis was found intraoperatively, these cases undergo partial hepatectomy based on the liver surgeon’s judgment. Fibrosis was defined as f3, and bridging fibrosis was defined as f4. Cirrhosis was defined according to the general rules for the clinical and pathological study of primary liver cancer set by the Liver Cancer Study Group of Japan [22].

Postoperative morbidity was assessed using the validated Clavien–Dindo classification system [23]. Serious complications were categorized as grades III–V and defined as morbidity requiring surgical or radiological intervention. Liver failure and hyperbilirubinemia were defined according to ISGLS grade B or C [24].

### Follow-up after hepatectomy

Patients were followed up at 3-month intervals. Patients underwent physical examination and serological examination, including alpha-fetoprotein (AFP) level, protein induced by vitamin K absence-II (PIVKA-II), and liver function. In addition, radiological examinations, including contrast-enhanced computed tomography (CT) scans and/or ultrasound sonography (US) or contrast-enhanced magnetic resonance imaging (MRI), were performed. Follow-up using these modalities after curative treatment at 3- or 4-month intervals was recommended by the Clinical Practice Guidelines for Hepatocellular Carcinoma 2017 [25].

### Statistical analyses

Differences in characteristic factors were evaluated by the Mann-Whitney *U* test for continuous variables or chi-square test for noncontinuous variables. Kaplan-Meier survival curves were compared by using the log-rank test. Overall survival (OS) and relapse-free survival (RFS) were evaluated. Prognostic factors were evaluated by these univariate analyses and multivariate analyses using the Cox proportional hazard model. We used JMP Pro 14.0.0 for Windows (SAS Institute, Cary, NC) for statistical analyses. A *p* value of < 0.05 was considered significant.

## Results

### Clinicopathological characteristics and operative variables

We divided the period between 2000 and 2017 and designated the period from 2000 to 2008 as the early period and the period from 2009 to 2017 as the late period. In the early period, group VE included 8 patients (2.1%, 8/390). In contrast, this group had 41 patients (10.1%, 41/406) in the late period.

In this cohort, the median survival time (MST) and 5-year OS rate in our 796 study patients were 103 months and 63%, respectively. The median RFS time was 20 months. The median length of hospital stay was 24 (9–386) days. The number of cases with HBV and HCV was 277 and 217, respectively. There were 15 patients with both HBV and HCV. There were 287 cases of NBNC (NonBNonC)-HCC. The median follow-up period of the whole cohort was 39 months. These were 24 months, 38 months, and 45 months (VE vs. E vs. Y), respectively.

Univariate analysis showed that the proportion of patients who were HCV positive, without HBV and HCV (NBNC), was significantly increased in group VE compared with group Y (Table 1). In contrast, the proportion of patients who were HBV positive with portal venous invasion (PVI) and liver fibrosis was significantly reduced in group VE compared with group Y. Preoperative cholinesterase (ChE), serum albumin, and AFP levels were significantly reduced in group VE compared with group Y. The proportion of patients who were HBV positive and had liver fibrosis was significantly reduced in group VE compared with group E (Table 1). Other factors, including the proportion of patients who were HCV positive, were not different between groups VE and E.

**Table 1:** Clinicopathological characteristics of HCC

Characteristics	Group VE (*n* = 49)	Group E (*n* = 363)	Group Y (*n* = 384)	*p*; VE vs. E	*p*; VE vs. Y
Epidemiology					
Age (years old)	82 (80–92)	71 (65–79)	57 (33–64)	*< 0.01*	*< 0.01*
Sex, male to female (%)	80(39):20(10)	83(301):17(62)	84(321):16(63)	*0.56*	*0.48*
HBs-Ag positive (%)	6 (3)	19 (69)	53 (205)	*0.02*	*< 0.01*
HCV-Ab positive (%)	35 (17)	34 (122)	21 (80)	*0.88*	*0.02*
Both HBs-Ag and HCV-Ab positive (%)	0 (0)	1 (5)	3 (10)	*0.40*	*0.25*
NBNC (%)	59 (29)	46 (167)	23 (89)	*0.08*	*< 0.01*
Biochemical factors					
Child-Pugh score	5.2 ± 0.5	5.2 ± 0.5	5.2 ± 0.5	*0.50*	*0.38*
Platelets (10^4^/mm^3^)	18.4 ± 6.8	17.5 ± 10.8	17.0 ± 7.6	*0.54*	*0.20*
PA (%)	94.8 ± 13.5	92.5 ± 14.0	91.4 ± 13.9	*0.28*	*0.10*
ChE (IU/l)	230 ± 68	244 ± 71	262 ± 90	*0.22*	*0.01*
Albumin (g/dl)	3.9 ± 0.3	4.0 ± 0.4	4.1 ± 0.5	*0.06*	*0.04*
ICGR15 (%)	15.8 ± 8.2	16.7 ± 9.6	15.8 ± 11.5	*0.56*	*0.94*
AFP (ng/ml)	5.5 (0–60,961)	10.6 (0–378,718)	27.5 (1–5,986,980)	*0.05*	*< 0.01*
PIVKA-II (mAU/ml)	198 (10–217,422)	181 (8–664,680)	207 (2.3–928,799)	*0.95*	*0.98*
Tumor factors					
Tumor size (cm)	6.4 ± 3.5	5.7 ± 4.1	6.1 ± 5.2	*0.23*	*0.66*
Multiple tumor (%)	31 (15)	35 (128)	38 (144)	*0.52*	*0.34*
Differentiation, poor (%)	39 (19)	37 (134)	46 (175)	*0.80*	*0.36*
PVI (%)	14 (7)	21 (75)	34 (131)	*0.29*	*< 0.01*
HVI (%)	14 (7)	11 (41)	14 (52)	*0.54*	*0.88*
Fibrosis, f3/f4 (%)	16 (8)	40 (146)	55 (213)	*< 0.01*	*< 0.01*

*VE* very elderly group, *E* elderly group, *Y* young group, *HBs-Ag* HBs-antigen, *HCV-Ab* HCV-antibody, *NBNC* without HBV and HCV, *PA* prothrombin activity, *ICGR15* indocyanine green retention rate at 15 min, *AFP* alpha-fetoprotein, *PIVKA-II* protein induced by vitamin K absence-II, *PVI* portal venous invasion, *HVI* hepatic venous invasion

Table 2 shows the surgical procedures and outcomes in group VE, group E, and group Y. The median operative time in group VE was significantly reduced compared with groups E and Y. No significant differences in blood loss or postoperative morbidities were noted.

**Table 2:** Surgical procedure and outcomes

	Group VE (*n* = 49)	Group E (*n* = 363)	Group Y (*n* = 384)	*p*; VE vs. E	*p*; VE vs. Y
Surgical procedure					
Partial hepatectomy (%)	20 (10)	23 (85)	25 (95)	*0.63*	*0.50*
Subsegmentectomy or segmentectomy (%)	41 (20)	41 (149)	36 (140)	*0.97*	*0.55*
Bisegmentectomy or trisegmentectomy (%)	39 (19)	36 (129)	39 (149)	*0.65*	*0.99*
Operative outcome					
Median blood loss (ml) (range)	400 (0–2400)	380 (0–35,820)	375 (0–20,190)	*0.64*	*0.50*
Median operative time (min) (range)	288 (113–508)	327 (99–911)	312 (78–609)	*< 0.01*	*0.01*
Morbidity					
Total morbidities (%)	14 (7)	19 (68)	23 (88)	*0.44*	*0.16*
Pleural effusion (%)	4 (2)	4 (15)	7 (27)	*0.98*	*0.43*
Ascites (%)	4 (2)	3 (12)	5 (19)	*0.77*	*0.79*
Postoperative bleeding (%)	2 (1)	2 (7)	4 (17)	*0.95*	*0.43*
Bile leakage (%)	8 (4)	7 (26)	6 (22)	*0.80*	*0.49*
Hyperbilirubinemia (%)	2 (1)	2 (9)	4 (15)	*0.85*	*0.51*
Wound infection (%)	2 (1)	2 (8)	2 (7)	*0.94*	*0.91*
Pneumonia (%)	4 (2)	1 (4)	2 (8)	*0.10*	*0.38*
Ileus (%)	4 (2)	1 (5)	1 (4)	*0.16*	*0.08*
Postoperative stay (days) (range)	22 (14–308)	25 (11–386)	24 (9–176)	*0.17*	*0.43*
Mortality					
30 days (%)	0 (0)	0 (0)	0 (0)		
90 days (%)	0 (0)	0 (0)	0.3 (1)		

*VE* very elderly group, *E* elderly group, *Y* young group

### Recurrence site and treatment after recurrence

Regarding recurrence, 24 patients experienced recurrence (49%) with a median recurrence time of 11 months (3–68) in group VE. In group E, 231 patients experienced recurrence (64%) with a median recurrence time of 11 months (0.4–111). In group Y, 261 patients experienced recurrence (68%) with a median recurrence time of 9 months (0.2–197). The initial recurrence sites were not significantly different among the three groups (Table 3). Table 3 also shows the treatment methods used after recurrence. No cases of rehepatectomy or liver transplantation were performed in group VE. The frequency of treating patients with rehepatectomy was significantly reduced in group VE compared with group Y.

**Table 3:** Initial recurrence patterns and treatment for recurrence

	Group VE (*n* = 49)	Group E (*n* = 363)	Group Y (*n* = 384)	*p*; VE vs. E	*p*; VE vs. Y
Recurrence cases (%)	49 (24)	64 (231)	68 (261)	*0.04*	*< 0.01*
Median recurrence duration (months) (range)	11 (3–68)	11 (0.4–111)	9 (0.2–197)	*0.98*	*0.39*
Recurrence site	(*n* = 24)	(*n* = 231)	(*n* = 261)		
Liver (%)	75 (18)	83 (192)	82 (213)	*0.32*	*0.42*
Lung (%)	33 (8)	18 (41)	29 (75)	0.06	0.63
Adrenal glands (%)	8 (2)	2 (5)	5 (14)	*0.07*	*0.54*
Bone (%)	13 (3)	11 (25)	10 (27)	*0.80*	*0.74*
Treatment for recurrence	(*n* = 24)	(*n* = 231)	(*n* = 261)		
Rehepatectomy (%)	0 (0)	6 (15)	46 (119)	*0.19*	*< 0.01*
Liver transplantation (%)	0 (0)	0 (0)	3 (8)	*-*	*0.38*
RFA/MCT (%)	33 (8)	25 (57)	18 (46)	*0.35*	*0.06*
TACE (%)	63 (15)	52 (121)	47 (122)	*0.34*	*0.13*
Resection of metastases (%)	13 (3)	3 (7)	5 (13)	*0.02*	*0.12*
Systemic chemotherapy including molecular target drug (%)	17 (4)	23 (53)	33 (85)	*0.48*	*0.10*
Radiation (%)	17 (4)	12 (27)	16 (43)	*0.47*	*0.98*

*VE* very elderly group, *E* elderly group, *Y* young group, *RFA* radiofrequency ablation, *MCT* microwave coagulation therapy, transcatheter arterial chemoembolization

### Survival after hepatectomy

A total of 288 (36%) patients died; 237 (82%) were cancer-related deaths. Five (1.7%) patients with controlled HCC experienced liver failure-related deaths. In total, 1 (0.3%) death was related to the surgical procedure (posthepatectomy liver failure); 46 (16%) deaths were classified as other disease-related deaths.

There were 11 (22%) deaths in group VE, 120 (33%) deaths in group E, and 157 (41%) deaths in group Y. The cause of death and the breakdown among the three groups were as follows (group VE vs. group E vs. group Y): cancer-related, 7 (64%) vs. 90 (75%) vs. 140 (89%) (*p* = 0.41, 0.01); liver failure not related to cancer, 0 (0%) vs. 3 (2.5%) vs. 2 (1%) (*p* = 0.59, 0.70); and others, 4 (36%) vs. 27 (22.5%) vs. 15 (10%) (*p* = 0.30, < 0.01). Group VE showed significantly fewer cancer-related deaths and more non-cancer-related deaths than group Y. No significant differences in the cause of death were noted between groups VE and E.

### Prognostic factors of elderly patients with HCC

**Fig. 1:**
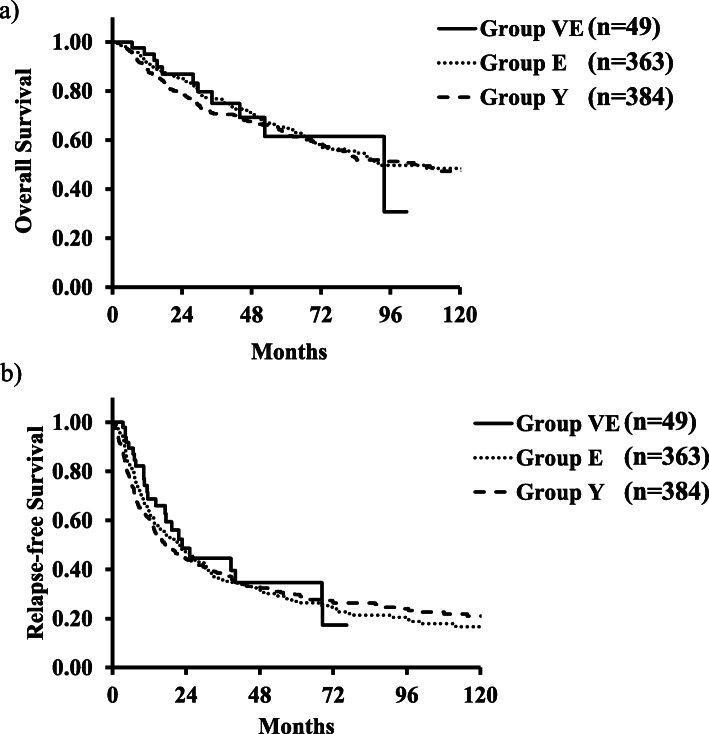
**a** Overall survival curves for patients with HCC among the VE group, E group, and Y group. **b** Relapse-free survival curves for patients with HCC among the VE group, E group, and Y group

The 5-year OS rate of group VE was 62%, and the rates of group E and group Y were 65% and 62%, respectively (*p* = 0.86). The median RFS times of groups VE, E, and Y were 22 months, 21 months, and 17 months, respectively (*p* = 0.65). Both the OS and RFS rates were not significantly different among the three groups (Fig. 1).

Table 4 presents the factors related to OS and RFS in group VE. The multivariate analysis indicated that a preoperative prothrombin activity (PA) < 80% and PIVKA-II ≥ 400 mAU/ml were unfavorable factors for OS and that PIVKA-II ≥ 400 mAU/ml and the presence of PVI, hepatic venous invasion (HVI), and fibrosis were unfavorable factors for RFS in group VE. Table 5 presents the factors related to OS and RFS in group E. The multivariate analysis indicated that preoperative ChE < 180 IU/l, AFP ≥ 20 ng/ml, tumor size ≥ 10 cm, and the presence of multiple tumors, PVI, and HVI were unfavorable factors for OS and that preoperative ChE < 180 IU/l, tumor size ≥ 10 cm, and the presence of multiple tumors, PVI, and HVI were unfavorable factors for RFS in group E. Table 6 presents the factors related to OS and RFS in group Y. Multivariate analysis indicated that AFP ≥ 20 ng/ml, the presence of multiple tumors, poor differentiation, PVI and HVI, and operative blood loss ≥ 400 ml were unfavorable factors for OS and that a preoperative PA < 80%, albumin < 3.5 g/dl, AFP ≥ 20 ng/ml, tumor size ≥ 10 cm, and the presence of multiple tumors, poor differentiation, and PVI were unfavorable factors for RFS in group Y.

**Table 4:** Prognostic factors for survival and recurrence of very elderly patients

	Overall survival	Relapse-free survival		
Characteristics	Univariate (*p*)	Multivariate (*p*) (hazard ratio) (95% CI)	Univariate (*p*)	Multivariate (*p*) (hazard ratio) (95% CI)
Epidemiology				
Sex, male	*0.64*		*0.29*	
HBs-Ag positive	*0.24*		*0.09*	
HCV-Ab positive	*0.28*		*0.94*	
NBNC	*0.07*		*0.63*	
Biochemical factors				
Platelets < 100,000/mm^3^	*0.50*		*0.42*	
PA < 80%	*< 0.01*	*0.02* (8.109) (1.227–53.587)	*0.57*	
ChE < 180 IU/l	*0.01*	*0.35* (2.021) (0.447–9.127)	*0.70*	
Albumin < 3.5 g/dl	*0.24*		*0.43*	
ICGR15 ≥ 15%	*0.06*		*0.77*	
AFP ≥ 20 ng/ml	*0.11*		*< 0.01*	*0.85* (0.911) (0.339–2.444)
PIVKA-II ≥ 400 mAU/ml	*< 0.01*	*0.03* (9.838) (1.220–79.309)	*0.01*	*0.02* (4.038) (1.147–14.211)
Tumor factors				
Tumor size ≥ 10 cm	*0.03*	*0.65* (0.707) (0.157–3.189)	*< 0.01*	*0.27* (0.424) (0.089–2.006)
Tumor number multiple	*0.93*		*0.04*	*0.20* (2.115) (0.672–6.657)
Macroscopic type except simple nodular	*0.42*		*0.03*	*0.05* (2.646) (0.982–7.127)
Histological factors				
Differentiation, poor	*0.35*		*0.02*	*0.45* (1.440) (0.552–3.757)
PVI	*0.75*		*0.01*	*0.01* (4.580) (1.342–15.629)
HVI	*0.24*		*0.02*	*< 0.01* (7.393) (1.923–28.424)
Fibrosis	*0.57*		*0.02*	*0.01* (3.483) (1.258–9.644)
Surgical factors				
FRLR < 50%	*0.03*	*0.81* (1.204) (0.245–5.911)	*0.17*	
Non-anatomical resection	*0.80*		*0.99*	
Blood loss ≥ 400 ml	*< 0.01*	*0.18* (5.000) (0.470–53.111)	*0.77*	

*PA* prothrombin activity, *ChE* cholinesterase, *ICGR15* indocyanine green retention rate at 15 min, *AFP* alpha-fetoprotein, *PIVKA-II* protein induced by vitamin K absence-II, *PVI* portal venous invasion, *HVI* hepatic venous invasion, *FRLR* future remnant liver rates

**Table 5:** Prognostic factors for survival and recurrence of elderly patients

	Overall survival	Relapse-free survival		
Characteristics	Univariate (*p*)	Multivariate (*p*) (hazard ratio) (95% CI)	Univariate (*p*)	Multivariate (*p*) (hazard ratio) (95% CI)
Epidemiology				
Sex, male	*0.62*		*0.66*	
HBs-Ag positive	*0.07*		*0.32*	
HCV-Ab positive	*0.52*		*0.87*	
NBNC	*0.12*		*0.63*	
Biochemical factors				
Platelets < 100,000/mm^3^	*0.93*		*0.35*	
PA < 80%	*0.17*		*0.04*	*0.40* (1.163) (0.813–1.665)
ChE < 180 IU/l	*< 0.01*	*< 0.01* (2.530) (1.684–3.800)	*< 0.01*	*< 0.01* (1.578) (1.125–2.214)
Albumin < 3.5 g/dl	*< 0.01*	*0.58* (1.163) (0.679–1.992)	*< 0.01*	*0.29* (1.282) (0.802–2.050)
ICGR15 ≥ 15%	*0.56*		*0.18*	
AFP ≥ 20 ng/ml	*< 0.01*	*0.03* (1.553) (1.041–2.315)	*< 0.01*	*0.55* (1.095) (0.811–1.480)
PIVKA-II ≥ 400 mAU/ml	*< 0.01*	*0.63* (0.905) (0.596–1.373)	*< 0.01*	*0.94* (0.989) (0.732–1.337)
Tumor factors				
Tumor size ≥ 10 cm	*< 0.01*	*0.01* (1.922) (1.128–3.274)	*< 0.01*	*0.01* (1.654) (1.102–2.484)
Tumor number multiple	*< 0.01*	*0.02* (1.559) (1.049–2.316)	*< 0.01*	*< 0.01* (2.223) (1.679–2.943)
Macroscopic type except simple nodular	*0.11*		*< 0.01*	*0.13* (1.249) (0.931–1.675)
Histological factors				
Differentiation, poor	*< 0.01*	*0.40* (1.179) (0.797–1.744)	*0.01*	*0.78* (0.959) (0.707–1.300)
PVI	*< 0.01*	*< 0.01* (2.653) (1.674–4.206)	*< 0.01*	*0.03* (1.460) (1.027–2.078)
HVI	*< 0.01*	*0.02* (1.889) (1.066–3.346)	*< 0.01*	*< 0.01* (1.980) (1.284–3.053)
Fibrosis	*0.42*		*0.60*	
Surgical factors				
FRLR ≥ 50%	*0.63*		*0.83*	
Non-anatomical resection	*0.20*		*0.06*	
Blood loss ≥ 400 ml	*0.02*	*0.97* (1.005) (0.674–1.500)	*0.04*	*0.91* (1.014) (0.772–1.333)

*PA* prothrombin activity, *ChE* cholinesterase, *ICGR15* indocyanine green retention rate at 15 min, *AFP* alpha-fetoprotein, *PIVKA-II* protein induced by vitamin K absence-II, *PVI* portal venous invasion, *HVI* hepatic venous invasion, *FRLR* future remnant liver rates

**Table 6:** Prognostic factors for survival and recurrence of young patients

	Overall survival	Relapse-free survival		
Characteristics	Univariate (*p*)	Multivariate (*p*) (hazard ratio) (95% CI)	Univariate (*p*)	Multivariate (*p*) (hazard ratio) (95% CI)
Epidemiology				
Sex, male	*0.42*		*0.26*	
HBs-Ag positive	*0.72*		*0.13*	
HCV-Ab positive	*0.18*		*0.80*	
NBNC	*0.38*		*0.13*	
Biochemical factors				
Platelets < 100,000/mm^3^	*0.84*		*0.34*	
PA < 80%	*0.03*	*0.27* (1.261) (0.833–1.908)	*< 0.01*	*0.01* (1.449) (1.063–1.974)
ChE < 180 IU/l	*< 0.01*	*0.13* (1.416) (0.901–2.225)	*< 0.01*	*0.79* (0.954) (0.666–1.366)
Albumin < 3.5 g/dl	*< 0.01*	*0.47* (1.215) (0.711–2.076)	*< 0.01*	*< 0.01* (1.834) (1.213–2.774)
ICGR15 ≥ 15%	*0.94*		*0.09*	
AFP ≥ 20 ng/ml	*< 0.01*	*< 0.01* (1.769) (1.242–2.520)	*< 0.01*	*0.02* (1.344) (1.029–1.755)
PIVKA-II ≥ 400 mAU/ml	*< 0.01*	*0.83* (1.045) (0.691–1.580)	*< 0.01*	*0.88* (0.977) (0.715–1.333)
Tumor factors				
Tumor size ≥ 10 cm	*< 0.01*	*0.78* (0.932) (0.570–1.526)	*< 0.01*	*0.01* (1.637) (1.122–2.388)
Tumor number multiple	*< 0.01*	*< 0.01* (1.674) (1.194–2.346)	*< 0.01*	*< 0.01* (1.704) (1.307–2.222)
Macroscopic type except simple nodular	*< 0.01*	*0.52* (0.880) (0.593–1.306)	*< 0.01*	*0.72* (0.947) (0.704–1.274)
Histological factors				
Differentiation, poor	*< 0.01*	*< 0.01* (1.818) (1.270–2.601)	*< 0.01*	*0.01* (1.419) (1.070–1.884)
PVI	*< 0.01*	*< 0.01* (1.904) (1.290–2.808)	*< 0.01*	*0.02* (1.467) (1.061–2.028)
HVI	*< 0.01*	*0.02* (1.718) (1.080–2.735)	*< 0.01*	*0.66* (1.089) (0.734–1.615)
Fibrosis	*0.18*		*0.05*	
Surgical factors				
FRLR ≥ 50%	*0.42*		*0.49*	
Non-anatomical resection	*0.79*		*0.89*	
Blood loss ≥ 400 ml	*< 0.01*	*0.01* (1.604) (1.108–2.323)	*< 0.01*	*0.15* (1.218) (0.927–1.601)

*PA* prothrombin activity, *ChE* cholinesterase, *ICGR15* indocyanine green retention rate at 15 min, *AFP* alpha-fetoprotein, *PIVKA-II* protein induced by vitamin K absence-II, *PVI* portal venous invasion, *HVI* hepatic venous invasion, *FRLR* future remnant liver rates

### Future remnant liver rates in pretty elderly patients who underwent hepatectomy

**Fig. 2:**
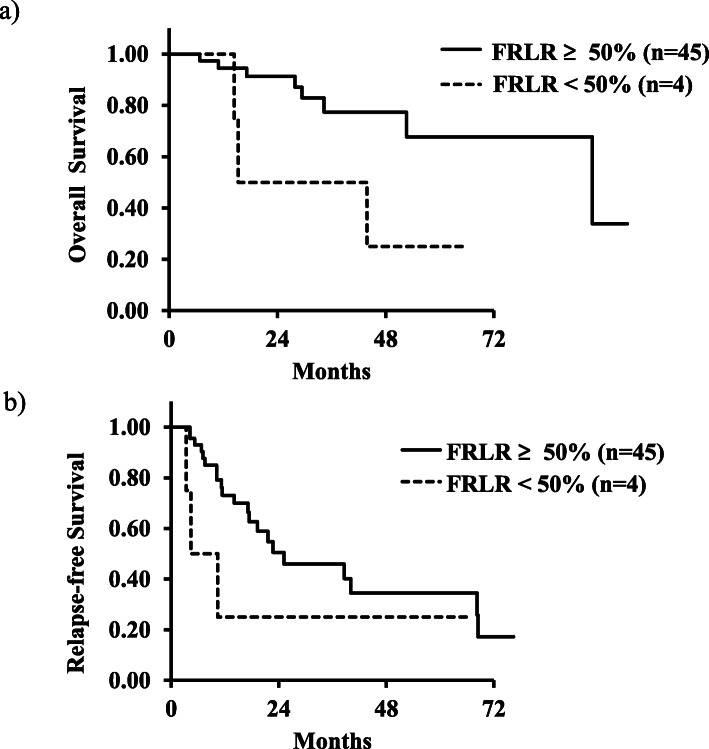
**a** Overall survival curves in group VE between the patients with an FRLR ≥ 50% and FRLR < 50%. **b** Relapse-free survival curves in group VE between the patients with an FRLR ≥ 50% and FRLR < 50%

We evaluated the future remnant liver rate (FRLR) in group VE. Patients with an FRLR ≥ 50% showed significantly more favorable survival than patients with an FRLR < 50% (*p* = 0.03). On the other hand, there was no significant difference in recurrence between patients with an FRLR ≥ 50% and FRLR < 50% (Fig. 2). Furthermore, there were no significant differences in survival between patients with an FRLR ≥ 50% and FRLR < 50% in groups E and Y (*p* = 0.63, 0.42; Tables 5 and 6).

## Discussion

Our present study indicated that the number of patients in group VE increased in the late period (2009–2017) and that there were an increased proportion of patients with HCV and patients without HBV and HCV.

The incidence of PVI and fibrosis was lower in group VE compared with group Y. The number of surgical procedures was not significantly different between groups. Group VE showed significantly fewer cancer-related deaths and more non-cancer-related deaths than group Y. The surgical outcomes and morbidities of group VE were almost the same as those of the other two groups. These three groups showed similar OS and RFS results. Regarding prognostic factors, tumor factors, such as tumor size and tumor number, less influenced the prognosis of patients in group VE compared with patients in groups E and Y.

According to the nationwide survey of HCC patients in Japan, the rate of nonviral HCC was 32.5% in 2015 [26]. In this study, the rate for group VE was 59%. This rate was high. Previous studies have reported that the number of elderly patients with HCC is increasing [27] and that elderly patients have higher rates of HCV or NBNC than nonelderly patients [18, 19, 27, 28], which is consistent with our results. HCV infections generally occur in adulthood in contrast to HBV infections, which are generally acquired through mother-child transmission [29]. The increased number of elderly NBNC-HCC patients is thought to be attributed to the fact that non-alcoholic fatty liver disease and non-alcoholic steatohepatitis-related HCC with metabolic syndromes are more likely to occur in elderly patients compared with young patients [30, 31]. Regarding liver function, elderly patients tend to develop HCC without cirrhosis or liver fibrosis [28]. Paradis et al. reported that HCC patients with metabolic syndromes showed less significant fibrosis than those without metabolic syndromes [30]. Tokushige et al. reported that cryptogenic HCC patients aged 80 years or older tended to develop HCC without cirrhosis [32]. Regarding oncological features, some reports have demonstrated an increased frequency of tumor encapsulation and lower vascular invasion in elderly patients compared with young patients [33, 34]. These results were consistent with our results. Katsuta et al. reported an age-related upregulation of the androgen and phosphatidylinositol 3-kinase pathways in tumor tissue and a downregulation of fibrosis-related pathways in noncancerous liver tissue [35]. Thus, compared with HCC in young patients, the characteristics of HCC in elderly patients could be somewhat different, such as a slightly lower degree of malignancy and relatively better liver function. In this study, prognostic factors, such as tumor size and tumor number, less influenced the prognoses of patients in group VE compared with patients in groups E and Y. This result might be explained by these biological differences. A previous study reported that HCC occurring at younger and elderly ages showed distinct oncogenic mechanisms by analyzing gene expression [36]. In addition, the livers of the elderly showed decreased liver regenerative capacity, altered metabolism functions, and immune response dysfunction, making them more susceptible to the development of chronic liver diseases [37]. Therefore, prognostic factors might differ among the different groups.

Liver function reserve might be more important in group VE compared with groups E or Y. Interestingly, patients with an FRLR ≥ 50% exhibited significantly more favorable survival than patients with an FRLR < 50% in group VE according to the univariate analysis. Furthermore, no significant differences in recurrence were noted. During hepatectomy for very elderly patients, surgeons might have to make a maximum effort to preserve the remnant liver as much as possible.

The prognoses of very elderly patients with HCC are under investigation. Many reports have claimed that the prognoses after hepatectomy do not differ between elderly patients and nonelderly patients [19, 27, 33]. Oishi et al. reviewed 23 papers and reported that the 5-year OS rates after hepatectomy in elderly HCC patients ranged from 26 to 75.9%, whereas rates in young patients ranged from 31.4 to 68%. Tsukioka et al. reported that in the early stage, patients with HCC aged 80 years or older had a poorer prognosis compared with nonelderly patients with HCC; however, there were no differences in all stages. Additionally, their study included not only hepatectomy but also other treatments [18]. Huang et al. reported that elderly patients had a better 5-year OS rate than younger patients (43.2% and 31.4%, respectively) [34]. In this study, the OS and RFS rates of very elderly patients were not different from those of elderly or young patients.

Hepatic resection is the main therapeutic method for HCC even in elderly patients. However, hepatectomy is a highly invasive surgical procedure with a high morbidity rate [11]. Therefore, the indications for hepatectomy in elderly patients with HCC represent an important consideration because these patients frequently have systemic comorbidities and low activities of daily living. Most previous studies have shown that morbidity rates after hepatectomy are not significantly different between elderly and nonelderly patients. These studies reported that the morbidities after hepatectomy in elderly patients ranged from 9 to 58% [34, 38, 39]. However, Ferrero et al. showed that elderly patients aged 70 years had lower complication rates after hepatectomy than young patients (23.4% vs. 42.4%), particularly in terms of liver failure (1.6% vs. 12.9%) [40]. The authors considered that this result was because elderly patients undergo more meticulous patient selection and less aggressive surgery compared with young patients. Kondo et al. reported that only the incidence of pneumonia after hepatectomy was significantly increased in elderly patients compared with young patients; however, the total complication rate and rates of other complications were not different between the groups [41]. In our study, these rates were not significantly different. Recent technological developments for hepatectomy and perioperative management have resulted in decreased mortality rates [11]. Hepatectomy should not be avoided in very elderly patients with HCC if the patients have a good general status. In our institute, we have empirically confirmed that cognitive function was well maintained and that patients were walking on their own at the outpatient consultation for the selection of elderly patients receiving hepatectomy.

Regarding treatment after recurrence, rehepatectomy was not performed in group VE in this cohort. This strategy was attributed to conservative patient selection. However, laparoscopic surgery might be a useful tool for rehepatectomy in very elderly patients.

The limitations of the study are as follows. First, the number of patients aged 80 years or older was small (*n* = 49). Second, elderly patients had a possibility of selection bias when they were referred from internal medicine.

## Conclusions

Tumor factors may less influence the prognoses of very elderly patients with HCC compared with patients younger than 80 years old, and liver function reserve might be important for the long-term survival of these elderly patients. Hepatectomy can be safely performed, even in very elderly patients, using a close evaluation. Hepatectomy should not be avoided in very elderly patients with HCC if patients have a good general status because these elderly patients with HCC have the same prognoses as nonelderly individuals with HCC.

## Data Availability

Not applicable

## Abbreviations

HCC: Hepatocellular carcinoma
TACE: Transcatheter arterial chemoembolization
HAI: Hepatic arterial infusion
HBs-Ag: HBs-antigen
HCV-Ab: HCV-antibody
ASA: American Society of Anesthesiologists
ICGR15: Indocyanine green retention test at 15 min
AFP: Alpha-fetoprotein
PIVKA-II: Protein induced by vitamin K absence-II
US: Ultrasound sonography
CT: Computed tomography
MRI: Magnetic resonance imaging
OS: Overall survival
RFS: Relapse-free survival
MST: Median survival time
NBNC: NonBNonC
PVI: Portal venous invasion
TACE: Transarterial chemoembolization
PA: Prothrombin activity
HVI: Hepatic venous invasion
ChE: Cholinesterase
FRLR: Future remnant liver rates

## References

[1] Bray F, Ferlay J, Soerjomataram I, Siegel RL, Torre LA, Jemal A (2018). Global cancer statistics 2018: GLOBOCAN estimates of incidence and mortality worldwide for 36 cancers in 185 countries. CA Cancer J Clin.

[2] Ahmed F, Perz JF, Kwong S, Jamison PM, Friedman C, Bell BP (2008). National trends and disparities in the incidence of hepatocellular carcinoma,1998-2003. Prev Chronic Dis.

[3] Wu J, Yang S, Xu K, Ding C, Zhou Y, Fu X (2018). Patterns and trends of liver cancer incidence rates in eastern and southeastern asian countries (1983-2007) and predictions to 2030. Gastroenterology..

[4] Liver Cancer Study Group of Japan (2019). 20th report of follow-up survey of primary liver cancer in Japan.

[5] E-Stat [internet]. https://www.e-stat.go.jp/en/stat-search/files?page=1&layout=datalist&toukei=00450011&tstat=000001028897&cycle=7&year=20160&month=0&tclass1=000001053058&tclass2=000001053061&tclass3=000001053072&result_back=1.

[6] Mhlw [internet]. https://www.mhlw.go.jp/english/database/db-hw/lifetb17/dl/lifetb17-01.pdf.

[7] Lee JH, Tak WY, Lee Y, Heo MK, Song JS, Kim HY (2017). Adjuvant immunotherapy with autologous dendritic cells for hepatocellular carcinoma, randomized phase II study. Oncoimmunology..

[8] Abdel Ghafar MT, Morad MA, El-Zamarany EA, Ziada D, Soliman H, Abd-Elsalam S (2020). Autologous dendritic cells pulsed with lysate from an allogeneic hepatic cancer cell line as a treatment for patients with advanced hepatocellular carcinoma: A pilot study. Int Immunopharmacol.

[9] Shindoh J, Makuuchi M, Matsuyama Y, Mise Y, Arita J, Sakamoto Y (2016). Complete removal of the tumor-bearing portal territory decreases local tumor recurrence and improves disease-specific survival of patients with hepatocellular carcinoma. J Hepatol.

[10] Phillip JM, Aifuwa I, Walston J, Wirtz D (2015). The Mechanobiology of Aging. Annu Rev Biomed Eng.

[11] Yokoo H, Miyata H, Konno H, Taketomi A, Kakisaka T, Hirahara N (2016). Models predicting the risks of six life-threatening morbidities and bile leakage in 14,970 hepatectomy patients registered in the National Clinical Database of Japan. Medicine(Baltimore).

[12] Tas F, Ciftci R, Kilic L, Karabulut S (2013). Age is prognostic factor affecting survival in lung cancer patients. Oncol Lett.

[13] Bechis SK, Carroll PR, Cooperberg MR (2011). Impact of age at diagnosis on prostate cancer treatment and survival. J Clin Oncol.

[14] Haymart MR (2009). Understanding the relationship between age and thyroid cancer. Oncologist..

[15] Zheng L, Wu C, Xi P, Zhu M, Zhang L, Chen S (2014). The survival and the long-term trends of patients with gastric cancer in Shanghai, China. BMC Cancer.

[16] Leff DR, Chen A, Roberts D, Grant K, Western C, Windsor AC (2007). Colorectal cancer in the young patient. Am Surg.

[17] Adami HO, Malker B, Holmberg L, Persson I, Stone B (1986). The relation between survival and age at diagnosis in breast cancer. N Engl J Med.

[18] Tsukioka G, Kakizaki S, Sohara N, Sato K, Takagi H, Arai H (2006). Hepatocellular carcinoma in extremely elderly patients: An analysis of clinical characteristics, prognosis and patient survival. World J Gastroenterol.

[19] Guo H, Wu T, Lu Q, Dong J, Ren YF, Nan KJ (2017). Hepatocellular carcinoma in elderly: Clinical characteristics, treatments and outcomes compared with younger adults. PLoS One.

[20] Kamiyama T, Nakanishi K, Yokoo H, Kamachi H, Tahara M, Yamashita K (2010). Perioperative management of hepatic resection toward zero mortality and morbidity: analysis of 793 consecutive cases in a single institution. J Am Coll Surg.

[21] Kawamura H, Kamiyama T, Nakagawa T, Nakanishi K, Yokoo H, Tahara M (2008). Preoperative evaluation of hepatic functional reserve by converted ICGR15 calculated from 99mTc-GSA scintigraphy. J Gastroenterol Hepatol.

[22] Liver cancer study group of Japan (eds), General rules for the clinical and pathological study of primary liver cancer. The 6^th^ edition. Kanehara & CO., LTD., Tokyo. 2015.

[23] Clavien PA, Barkun J, de Oliveira ML, Vauthey JN, Dindo D, Schulick RD (2009). The Clavien-Dindo classification of surgical complications: five-year experience. Ann Surg.

[24] Rahbari NN, Garden OJ, Padbury R, Brooke-Smith M, Crawford M, Adam R (2011). Posthepatectomy liver failure: a definition and grading by the International Study Group of Liver Surgery (ISGLS). Surgery..

[25] The Japan Society of Hepatology. Clinical Practice Guidelines for Hepatocellular Carcinoma 2017. https://www.jsh.or.jp/English/guidelines_en/Guidelines_for_hepatocellular_carcinoma_2017. Accessed 19 May 2020.

[26] Tateishi R, Uchino K, Fujiwara N, Takehara T, Okanoue T, Seike M (2019). A nationwide survey on non-B, non-C hepatocellular carcinoma in Japan: 2011-2015 update. J Gastroenterol.

[27] Oishi K, Itamoto T, Kohashi T, Matsugu Y, Nakahara H, Kitamoto M (2014). Safety of hepatectomy for elderly patients with hepatocellular carcinoma. World J Gastroenterol.

[28] Kinoshita A, Koike K, Nishino H (2017). Clinical features and prognosis of elderly patients with hepatocellular carcinoma not indicated for surgicali resection. Geriatr Gerontol Int.

[29] El-Serag HB (2012). Epidemiology of viral hepatitis and hepatocellular carcinoma. Gastroenterology..

[30] Paradis V, Zalinski S, Chelbi E, Guedj N, Degos F, Vilgrain V (2009). Hepatocellular carcinomas in patients with metabolic syndrome often develop without significant liver fibrosis: a pathological analysis. Hepatology..

[31] Ascha MS, Hanouneh IA, Lopez R, Tamimi TA, Feldstein AF, Zein NN (2010). The incidence and risk factors of hepatocellular carcinoma in patients with nonalcoholic steatohepatitis. Hepatology..

[32] Tokushige K, Hashimoto E, Horie Y, Taniai M, Higuchi S (2015). Hepatocellular carcinoma based on cryptogenic liver disease: The most common non-viral hepatocellular carcinoma in patients aged over 80 years. Hepatol Res.

[33] Yeh CN, Lee WC, Jeng LB, Chen MF (2004). Hepatic resection for hepatocellular carcinoma in elderly patients. Hepatogastroenterology..

[34] Huang J, Li BK, Chen GH, Li JQ, Zhang YQ, Li GH (2009). Long-term outcomes and prognostic factors of elderly patients with hepatocellular carcinoma undergoing hepatectomy. J Gastrointest Surg.

[35] Katsuta E, Tanaka S, Mogushi K, Matsumura S, Ban D, Ochiai T (2014). Age-related clinicopathologic and molecular features of patients receiving curative hepatectomy for hepatocellular carcinoma. Am J Surg.

[36] Wang HW, Hsieh TH, Huang SY, Chau GY, Tung CY, Su CW (2013). Forfeited hepatogenesis program and increased embryonic stem cell traits in young hepatocellular carcinoma (HCC) comparing to elderly HCC. BMC Genomics.

[37] Allaire M, Gilgenkrantz H (2020). The aged liver: Beyond cellular senescence. Clin Res Hepatol Gastroenterol.

[38] Ueno M, Hayami S, Tani M, Kawai M, Hirono S, Yamaue H (2014). Recent trends in hepatectomy for elderly patients with hepatocellular carcinoma. Surg Today.

[39] Hamaoka M, Kobayashi T, Ishiyama K, Ohira M, Tahara H, Kuroda S (2017). Evaluation of the risk factors and prognostic factors of hepatectomy for hepatocellular carcinoma in patients aged 80 years or more. J Hepatobiliary Pancreat Sci.

[40] Ferrero A, Vigano L, Polastri R, Ribero D, Lo Tesoriere R, Muratore A (2005). Hepatectomy as treatment of choice for hepatocellular carcinima in elderly cirrhotic patients. World J Surg.

[41] Kondo K, Chijiiwa K, Funagayama M, Kai M, Otani K, Ohuchida J (2008). Hepatic resection is justified for elderly patients with hepatocellular carcinoma. World J Surg.

